# Glycemic responses to low-intensity, short-duration aerobic exercise sessions in hospitalized women with gestational diabetes: a pilot study

**DOI:** 10.20945/2359-4292-2026-0104

**Published:** 2026-09-12

**Authors:** Edilson Vitória Nunes, Marcelo Zanusso Costa, Lidiane Pozza Costa, Gabriela de Lemos Uliano, Cristine Lima Alberton, Jorge Luiz de Brito Gomes, Juliano Boufleur Farinha

**Affiliations:** 1 Escola Superior de Educação Física e Fisioterapia, Universidade Federal de Pelotas, Pelotas, RS, Brasil; 2 Hospital Escola da Universidade Federal de Pelotas, HU Brasil, Pelotas, RS, Brasil; 3 Universidade Federal do Vale do São Francisco, Petrolina, PE, Brasil

**Keywords:** Gestational diabetes, blood glucose, postprandial period, aerobic exercise, hospitalization

## Abstract

**Objective:**

This study aimed to investigate the effects of low-intensity aerobic exercise performed once (30 min; A30) or twice a day (15+15 min; A15+15) on pre- and postprandial capillary blood glucose (CBG) levels on the day of the exercise session (D1) and the following day (D2) in hospitalized women with gestational diabetes mellitus (GDM).

**Subjects and methods:**

In this crossover study, nine participants adhered to a standard hospital diet and completed 2-day trials under three conditions: A30 (30 min in the afternoon), A15+15 (15 min in the morning and afternoon), and resting (REST). Participants completed the REST condition first. Preprandial (immediately before meals) and postprandial (60 min after main meals) CBG levels were measured. Exercise sessions comprised cycling at 50%–65% of maximal heart rate using a friction-based pedal. Data were analyzed using generalized estimating equations with Bonferroni post hoc analysis.

**Results:**

Pooled data from D1 and D2 (six measurements each day) showed lower CBG levels in the A15+15 condition (5.7 ± 0.3 mmol/L) compared to REST (6.3 ± 0.6 mmol/L) (~-9.5%) (*p* = 0.001); however, this finding carried limited clinical significance. There were no differences among the three experimental conditions when considering the same pre- and postprandial time points.

**Conclusion:**

Short-duration, low-intensity aerobic exercise sessions (A15+15) attenuated overall daily (pre- and postprandial) glycemia in hospitalized women with GDM. This attenuation was only observed in the pooled data, as glucose levels did not differ at specific postprandial time points.

## INTRODUCTION

Gestational diabetes mellitus (GDM) is characterized by carbohydrate intolerance diagnosed during pregnancy. It represents a significant health concern that increases the risk of unfavorable perinatal outcomes (^1^). The rising prevalence of GDM parallels the obesity epidemic among women of childbearing age (^2^). Maintaining maternal glycemic control is associated with lower rates of miscarriage, congenital malformations, fetal growth restriction, fetal macrosomia, and neonatal hypoglycemia.

GDM affects approximately 17% of pregnancies globally (^3^,^4^). Affected women face an increased risk of subsequently developing obesity, type 2 diabetes mellitus (T2DM), recurrent GDM, and cardiovascular morbidity (^5^). Data from the Brazilian Pelotas cohort study demonstrated that only about one-sixth of pregnant women engage in leisure-time physical activity (^6^). Initiating an exercise routine during pregnancy remains safe for most women, with few contraindications (^7^,^8^). Because patients typically interact more frequently with the healthcare system during pregnancy, this period provides an opportune window for establishing lasting lifestyle modifications.

Diet and physical exercise represent the first-line treatments for GDM, supplemented by pharmacological therapy when necessary (^9^). Unlike insulin or oral antidiabetic agents, exercise incurs minimal costs and avoids medication-related side effects. Some health guidelines suggest dividing daily exercise into smaller blocks to make daily goal more achievable (^7^,^10^). In this regard, recent evidence has shown that pregnant women with GDM who engaged in 20 minutes (min) of interval walking after each main meal (breakfast, lunch, and dinner) over a 4-day period presented reduced glucose excursions at specific time points (^3^). Although lifestyle modifications can mitigate perinatal risks, some sedentary women may still require hospitalization to correct their glycemic values.

Most women with GDM fail to meet recommended physical activity levels due to time constraints, tiredness, household work, raising children, safety concerns, and/or cultural beliefs that pregnancy requires absolute resting (^10^–^12^). Consequently, relatively short-duration (≤ 20–30 min), low-intensity sessions offer a practical strategy to increase exercise adherence among patients experiencing fatigue and limited time. Intermittent walking training has also been shown to improve cardiometabolic risk factors, including blood lipids, blood pressure, and body composition, in women with obesity (^13^). Exercise is an adjuvant tool for improving the metabolic health of women and their fetus, in addition to potentially reducing healthcare costs by shortening hospital stays. Because current guidelines recommend 60–150 min of aerobic exercise per week for women with GDM, clinicians must evaluate different continuous aerobic exercise (CAE) regimens of equivalent daily volume (e.g., 30 min) to increase exercise compliance in these individuals’ daily routines. Therefore, we aimed to investigate the effects of low-intensity, short-duration aerobic exercise sessions performed once daily (30 min) or divided into two 15-min bouts (15+15 min) on pre- and postprandial glucose levels during the day of exercise and the following day in hospitalized women with GDM.

## SUBJECTS AND METHODS

### Participants

We recruited participants from June 2022 to May 2023 at a tertiary public assistance and delivery hospital in southern Brazil. After screening medical and nursing records at the hospital’s Division of Obstetrics and Gynecology, we invited potentially eligible hospitalized patients to participate. Interested individuals were fully informed about the experimental procedures and provided informed consent. The hospital operates under an open-door policy, focusing on the care of women with high-risk pregnancies, including preterm labor, endocrine disorders, infectious or hemodynamic complications, and other disturbances. All participants received outpatient GDM management at specialized ambulatory care (endocrinology and metabolism, obstetrics, and nursing services) before and after this hospital admission. This study was approved by the local Ethics Committee (registration no. 31570720.5.0000.5317) and registered in the Brazilian Registry of Clinical Trials (ID: RBR-3xsnyd).

The inclusion criteria comprised (a) presence of GDM (fasting glycemia 5.1–6.9 mmol/L and/or 2-h glycemia 8.5–11.0 mmol/L following a 75-g oral glucose tolerance test) (^14^) in the current pregnancy; (b) gestational age between 26 and 37 weeks (third trimester); (c) age between 18 and 40 years; (d) body mass index between 20 and 45 kg/m^2^; (e) a protein/creatinine ratio ˂ 30 mg/mmol; and (f) physical inactivity (no planned and structured exercise in the previous three months). We excluded patients with type 1 diabetes mellitus or T2DM, infectious diseases, or significant impairment of renal or hepatic function. We also excluded participants with relative contraindications to exercise (severe anemia, uncontrolled hypertension or preeclampsia, orthopedic limitations, and heavy smoking) or absolute contraindications (hemodynamically significant heart disease, incompetent cervix/cerclage, persistent second- or third-trimester bleeding, placenta previa, premature labor during the current pregnancy, or ruptured embryonic membranes) (^7^).

### Study design

This crossover study was designed to minimize alterations to the hospital’s routine care. Participants acted as their own controls in this one-group repeated-measures counterbalanced design, enabling comparisons between a resting condition and two CAE protocols. Participants were instructed to only consume the diet provided by the Nutrition Service during hospitalization, consisting of three main meals and three snacks. Medical staff prescribed pharmacological treatments for GDM (i.e., insulin and metformin) during hospitalization according to established recommendations (^15^).

Participants completed 2-day trials under three conditions separated by at least 48 hours: 30 min of aerobic exercise in the afternoon (A30), 15 min of aerobic exercise in the morning and 15 min in the afternoon (A15+15), or a resting condition (REST). Day 1 (D1) was considered the exercise day, and Day 2 (D2) was the subsequent non-exercise day. Despite a systematic bias, REST was the first condition accomplished, since exams (e.g., cervical dilation, screening for preeclampsia, ultrasonography imaging, hepatic, thyroid, and renal biochemical parameters) were done or asked by medical staff in the first hours/days of hospitalization for complete screening of each case. After medical staff establishing the clinical management plan (including glycemic control), we randomized the exercise protocols (A30 and A15+15) using a simple coin toss (1:1 ratio). Only participants who completed at least two experimental conditions (REST plus A30 or REST plus A15+15) before discharge were considered in the statistical analysis.

**Figure 1:**
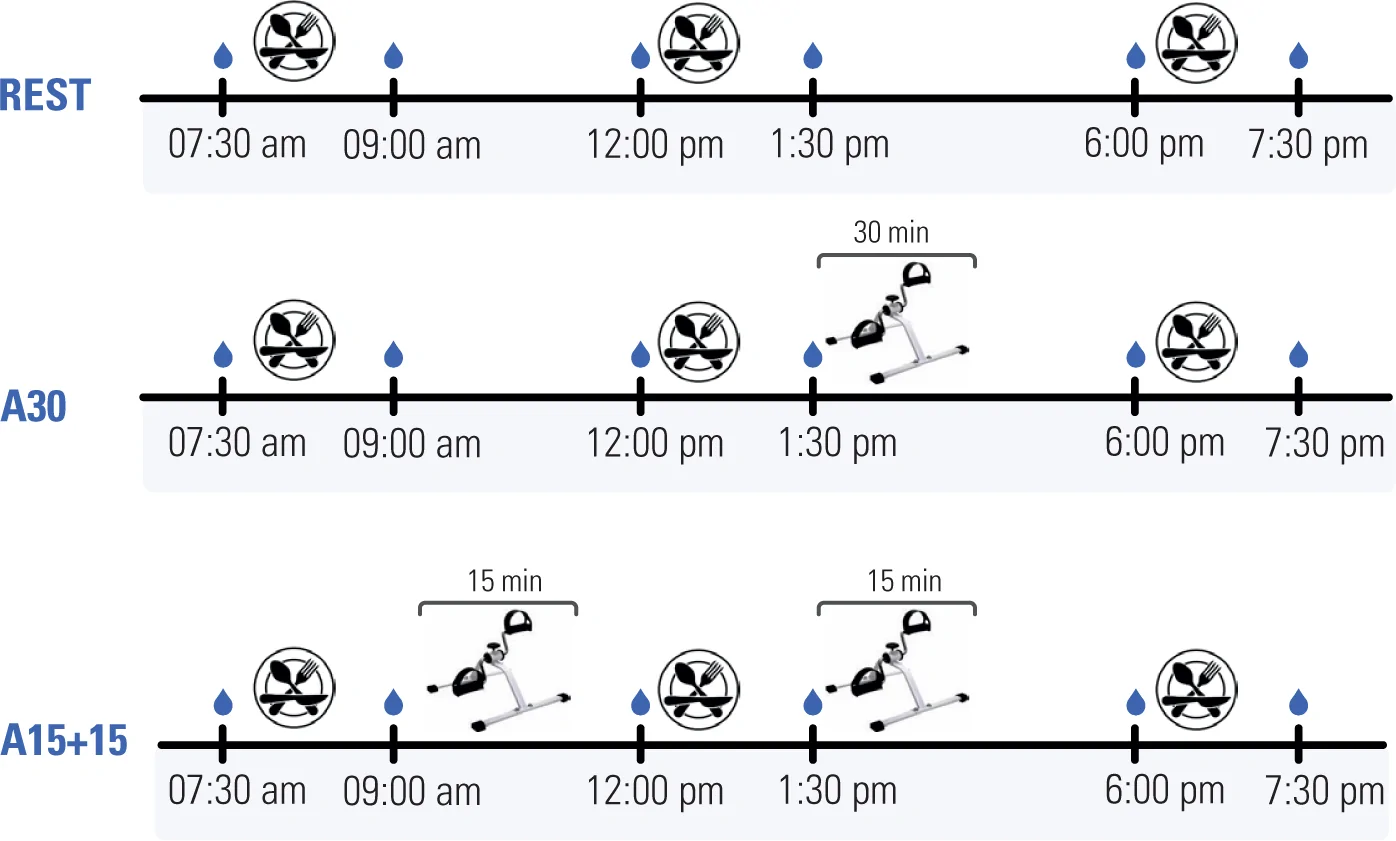
Study design. 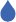: Capillary blood glucose measurement; 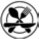: meal; 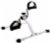: cycling.

REST: resting condition; A15+15: 15 min of aerobic exercise in the morning and 15 min in the afternoon; A30: 30 min of aerobic exercise in the afternoon.

In A30 condition, exercise sessions were conducted at 1:30 pm; in the A15+15 condition, sessions began at 9:00 am and 1:30 pm. Participants performed the A30 and A15+15 protocols approximately 60 min after finishing breakfast or lunch, matching the typical time of peak glycemia (^4^). Throughout their hospital stay, we measured patients’ capillary blood glucose (CBG) levels at the following time points: 7:30 am (fasting), 9:00 am (~60 min after finishing breakfast), 12:00 pm (immediately before lunch), 1:30 pm (~60 min after finishing lunch), 6:00 pm (immediately before dinner), and 7:30 pm (~60 min after finishing dinner). Hospitalized patients also received snacks between breakfast and lunch, between lunch and dinner, and between dinner and bedtime. CBG levels were also measured immediately before and after each exercise session. Figure 1 presents the study design for the exercise day (D1).

### Experimental conditions

The following experimental conditions were employed:

- **A30:** Aerobic exercise for 30 min in the afternoon.
- **A15+15:** Aerobic exercise for 15 min in the morning and 15 min in the afternoon.
- **REST:** Resting, in which participants did not exercise.

Participants performed all exercise sessions at 50%–65% of their maximal heart rate (HR_max_) (^7^,^16^) using a friction-based pedal device (seated cycling) in their hospital rooms. Exercise physiologists supervised the sessions to continuously adjust pedal rate or resistance to maintain target heart rate (HR), monitor intensity, and provide constant motivation. Participants wore a finger pulse oximeter during exercise to measure oxygen saturation and HR every 3 min. Water was provided *ad libitum* during the exercise sessions.

### Glucose measurements and diet control

CBG levels were measured using a handheld glucose meter (On Call^®^ Plus II, ACON, USA) at the time points detailed above. Participants would ingest a 20-g carbohydrate gel if their CBG levels were ≤ 4 mmol/L before starting any exercise protocol. Participants were strongly encouraged to only consume the hospital’s diet to minimize nutritional bias. Total energy and carbohydrate intake provided by the Nutrition Service were compared each day of the experimental period.

### Statistical analysis

Glycemic response (primary outcome) and secondary outcome variables were analyzed using generalized estimating equations (GEE). We based model selection for the best overall fit on the lowest Quasi information criterion, employing an unstructured working correlation matrix (robust estimator), a linear or gamma distribution (according to lower Quasi information criterion value), and an identity link function. The main effects were determined by Wald’s χ^2^ statistic and a sequential Bonferroni post hoc test was conducted for pairwise comparisons.

GEE analyses were performed to examine the main effects of the three conditions (A30, A15+15, REST) and six time points (7:30 am, 12:00 pm, and 6:00 pm on D1 and D2; or 9:00 am, 1:30 pm, and 7:30 pm on both D1 and D2), as well as their respective interaction effects. Separate GEE analyses were conducted for preprandial and postprandial data. For dietary variables, insulin and metformin dosages, and mean CBG levels across days, GEE analyses assessed the main effects of the three conditions (A30, A15+15, REST) and two time points (D1 and D2), as well as their respective interaction effects. An additional GEE analysis compared CBG levels before and after each exercise session (A30, A15+15 in the morning, and A15+15 in the afternoon). Comparisons among the exercise conditions (A30, A15+15, and REST) for HR and CBG delta changes (after minus before CBG values) were analyzed using a general linear model. This model considered a normal distribution, and the main effects were assessed using pairwise comparisons with sequential Bonferroni post hoc tests. All statistical analyses were performed using the Statistical Package for the Social Sciences (IBM SPSS v. 20.0, USA) with an alpha level of 5%. Results are presented as the mean ± standard deviation.

## RESULTS

Thirteen patients with GDM who met the eligibility criteria were invited to participate in the study (convenience sample). Two women declined to participate due to the perceived demands of the protocol, and two women were discharged from the hospital before completing at least one entire exercise protocol (A30 or A15+15). Descriptive data for the remaining participants (n = 9) and their childbirths are shown in Table 1.

**Table 1.** Descriptive characteristics of the sample (n = 9) in their current pregnancy

Variable	Mean ± SD or absolute frequency
Gestational age at study entry (weeks)	32.2 ± 3
Age (years)	32.1 ± 3.3
Body mass index (kg/m^2^)	31.9 ± 6
Body weight (kg)	84.2 ± 13.2
Glycated hemoglobin (%)	5.4 ± 0.6
Systolic blood pressure (mmHg)	122 ± 7.8
Diastolic blood pressure (mmHg)	68.2 ± 6.6
Use of neutral protamine Hagedorn insulin (n)	4
Use of regular insulin (n)	2
Use of extended-release metformin (n)	7
Gestational age at delivery (weeks)	37.6 ± 0.6
Cesarean/vaginal birth (n)	8/1

SD: standard deviation.

Participants were ~32 years old, had 3.0 ± 1.2 total (prior and current) pregnancies, and delivered at a mean gestational age of ~37.6 weeks. The average length of hospital stay was 8.4 ± 3.0 days. Eight participants did not give birth during their stay at the hospital (i.e., they were discharged and readmitted later for delivery). There were no multifetal gestations, and no infants were born weighing > 4 kg (macrosomia). Nine participants (100%) completed the REST condition, while the A30 and A15+15 conditions were each completed before hospital discharge by seven participants (77.8%).

Total daily energy intake provided by the Nutrition Service during hospitalization and experimental conditions on D1 and D2, respectively, was: 1628.0 ± 154.2 and 1628.0 ± 154.2 kcal (REST); 1605.7 ± 82.9 and 1669.3 ± 121.1 kcal (A15+15); and 1606.3 ± 145.7 and 1666.8 ± 103.6 kcal (A30). In this line, provided carbohydrate intake on D1 and D2, respectively, was: 176.6 ± 19.2 and 176.6 ± 19.2 g (REST); 184.9 ± 14.6 and 175.7 ± 13.0 g (A15+15); and 176.4 ± 16.2 and 182.6 ± 12.2 g (A30). Regarding a possible nutritional bias, there were no significant effects for total energy (*p* = 0.963 for condition, *p* = 0.174 for time/day, and *p* = 0.397 for interaction effects) or carbohydrate (*p* = 0.778 for condition, *p* = 0.753 for time/day, and *p* = 0.120 for interaction effects) intake across days and experimental conditions.

During the hospital stay, seven women used extended-release metformin, four received neutral protamine Hagedorn insulin, and two received regular insulin. Mean daily insulin dosages on D1 and D2, respectively, were: 1.1 ± 2.2 and 4.9 ± 5.4 IU (REST); 7.3 ± 7.9 and 8.0 ± 8.8 IU (A15+15); and 3.6 ± 5.0 and 5.8 ± 6.3 IU (A30). No differences were observed in condition (*p* = 0.264) or interaction (*p* = 0.379); however, a significant time/day effect was found (χ^2^ = 5.696, *p* = 0.017). These results indicate higher mean insulin dosages (pooled data from A30, A15+15, and REST) in D2 (6.2 ± 5.6 UI) than in D1 (4.0 ± 4.3 UI). Mean extended-release metformin dosages on D1 and D2, respectively, were: 1.0 ± 0.8 and 1.1 ± 0.5 g (REST); 0.6 ± 0.5 and 0.6 ± 0.5 g (A15+15); and 0.8 ± 0.8 and 0.6 ± 0.8 g (A30). No significant main effects of condition (*p* = 0.057) or time/day (*p* = 0.651), nor interaction effects (*p* = 0.698), were found.

CBG values measured immediately before and after each exercise session, respectively, were: 7.8 ± 0.8 and 7.0 ± 1.3 mmol/L (A15+15 in the morning); 6.2 ± 0.5 and 5.4 ± 0.5 mmol/L (A15+15 in the afternoon); and 6.4 ± 0.8 and 6.0 ± 0.8 mmol/L (A30). Significant main effects were observed for condition (χ^2^ = 57.772, *p* < 0.001) and time (χ^2^ = 27.492, *p* < 0.001), with no significant interaction (*p* = 0.300). CBG delta changes (after minus before CBG values) were also observed: -0.5 ± 1.3 mmol/L (A15+A15 in the morning), -0.7 ± 0.3 mmol/L (A15+A15 in the afternoon), and -0.5 ± 0.5 mmol/L (A30), with no differences among conditions (*p* = 0.394). Pooled pre- and post-exercise data revealed significantly higher CBG levels during the morning A15+15 session (7.4 ± 1.1 mmol/L) compared with both the afternoon A15+15 session (5.8 ± 0.5 mmol/L) and the A30 session (6.2 ± 0.8 mmol/L). In addition, reduced CBG levels (pooled data from A30, A15+15, REST) were found immediately after experimental conditions (6.1 ± 0.8 mmol/L) compared to those immediately before (6.7 ± 0.5 mmol/L). No hypoglycemic episodes (CBG ≤ 4.0 mmol/L) occurred immediately before, during, or immediately after exercise sessions, even among participants receiving insulin therapy. No exercise-related adverse events were reported. Mean HR values were similar (*p* = 0.444) across experimental conditions (~105 bpm; ~56% HR_max_).

**Figure 2:**
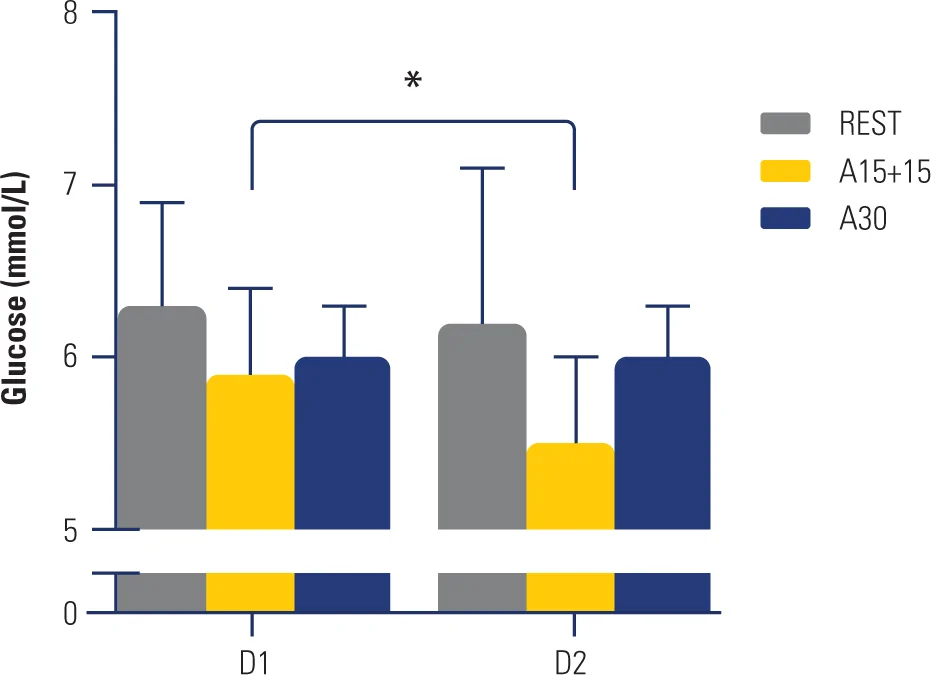
Mean capillary blood glucose levels across both days (six measurements per day).

REST: resting condition; A15+15: 15 min of aerobic exercise in the morning and 15 min in the afternoon; A30: 30 min of aerobic exercise in the afternoon; D1: Day 1; D2: Day 2. A significant condition effect was observed between A15+15 and REST. *Statistical difference from REST.

Figure 2 depicts mean CBG levels across D1 and D2 (pooled data from six time points each day). Mean CBG values observed on D1 and D2, respectively, were: 6.3 ± 0.6 and 6.2 ± 0.9 mmol/L (REST); 5.9 ± 0.5 and 5.5 ± 0.5 mmol/L (A15+15); and 6.0 ± 0.3 and 6.0 ± 0.3 mmol/L (A30). There were no differences in time/day (*p* = 0.09) or interaction (*p* = 0.320), albeit a condition effect was found (χ^2^ = 15.078, p = 0.001). These results indicate that lower (~-9.5%) CBG levels (pooled data from D1 and D2, six measurements each day) were observed in A15+15 (5.7 ± 0.3 mmol/L) than in REST (6.3 ± 0.6 mmol/L).

**Figure 3:**
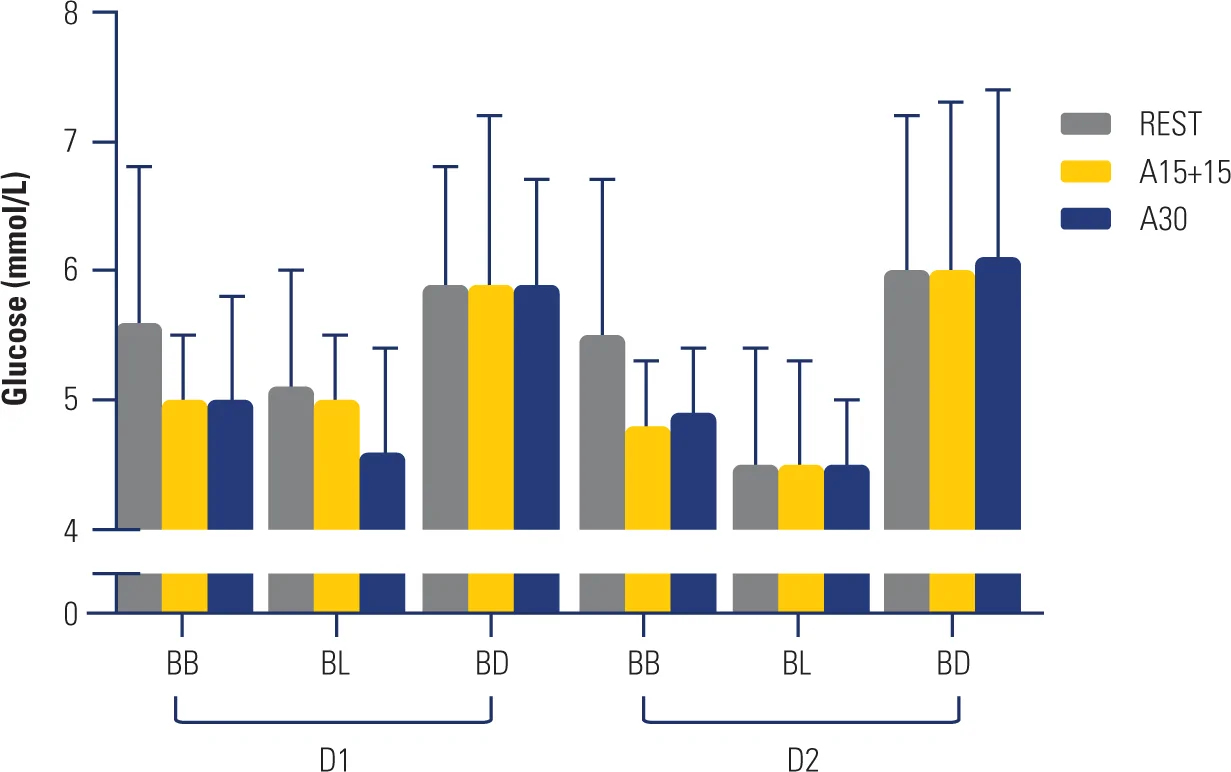
Preprandial capillary blood glucose levels under experimental conditions.

REST: resting condition; A15+15: 15 min of aerobic exercise in the morning and 15 min in the afternoon; A30: 30 min of aerobic exercise in the afternoon; BB: before breakfast; BL: before lunch; BD: before dinner; D1: Day 1; D2: Day 2.

Regarding preprandial CBG levels, GEE revealed a significant main effect of time (pooled data from six time points across D1 and D2; χ^2^ = 78.260, *p* < 0.001), with no significant main effect of condition (*p* = 0.662) or interaction (*p* = 0.805). Pairwise comparisons of pooled data from the three experimental conditions showed significant differences between before breakfast (BB) on D1 and before lunch (BL) on D2 (*p* = 0.011). Preprandial CBG levels were significantly higher before dinner (BD) on D1 compared with BL on D1 (*p* = 0.004), BB on D2 (*p* = 0.003), and BL on D2 (*p* < 0.001). Similarly, CBG values were higher BD on D2 compared with BB (*p* = 0.023) and BL (*p* < 0.001) on D1, as well as BB (*p* = 0.001) and BL (*p* < 0.001) on D2. No significant differences were observed among the three experimental conditions when considering the same time points (Figure 3).

**Figure 4:**
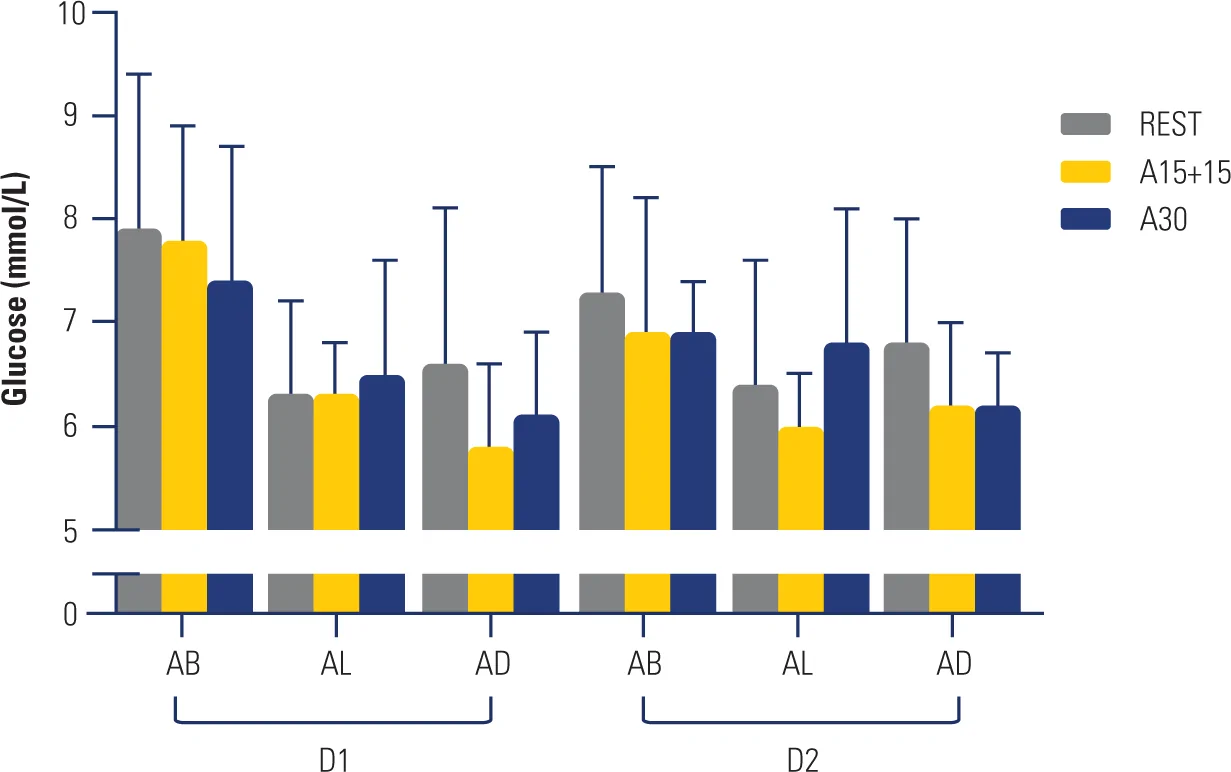
Postprandial capillary blood glucose levels under experimental conditions.

REST: resting condition; A15+15: 15 min of aerobic exercise in the morning and 15 min in the afternoon; A30: 30 min of aerobic exercise in the afternoon; AB: after breakfast; AL: after lunch; AD: after dinner; D1: Day 1; D2: Day 2.

Analysis of postprandial CBG levels via GEE revealed a significant main effect of time (pooled data from six time points across D1 and D2; χ^2^ = 52.678, *p* < 0.001), while condition (*p* = 0.613) and interaction (*p* = 0.816) effects were not observed. Pairwise comparisons of pooled data from the three experimental conditions indicated that CBG levels were significantly higher after breakfast (AB) on D1 compared with after lunch (AL) and after dinner (AD) on D1 (*p* < 0.001 for both), as well as AL and AD on D2 (*p* < 0.001 for both). Moreover, higher CBG values were observed AB on D2 compared with AD on D1 (*p* = 0.007) and AD on D2 (*p* = 0.047). Postprandial CBG levels peak after breakfast. Notably, no significant differences were observed among the three experimental conditions when considering the same time points (Figure 4).

## DISCUSSION

The main findings of this study include (^1^) lower CBG levels (pooled data from D1 and D2, representing the sum of pre- and postprandial glycemia each day) in the A15+15 condition compared with REST (~-9.5%), despite limited clinical relevance, and (^2^) no differences being found among the three experimental conditions at identical pre- and postprandial time points. To the best of our knowledge, this is the first study to compare the effects of low-intensity, relatively short-duration (30 min) CAE sessions, whether performed once (30 min) or divided into two 15-min bouts (15+15 min) on pre- and postprandial glycemia in hospitalized women with GDM.

In the study by Andersen and cols. (^3^), following a 4-day period, the authors found lower glycemic excursions during diurnal hours in the first three days of the protocol in a sample comprising non-insulin treated GDM women completing 20-min moderate-intensity interval walking (alternating 3-min slow and fast intervals) three times a day (after each main meal, comprising 60 min/day) and with identical diets. Recently, Christie and cols. (^4^) and Brislane and cols. (^5^) compared the effects of three 10-min postprandial CAE walking sessions (totaling 30 min/day) with a single 30-min CAE walking session over 3-5 days. Neither study found differences in postprandial glycemia between the moderate-intensity exercise protocols and baseline conditions (^4^,^5^). However, women with GDM who completed the three 10-min postprandial walking sessions accumulated more daily minutes of prescribed physical activity than those in the 30-min CAE condition (^4^). Similarly, our study found no differences among the exercise conditions and REST at matched postprandial time points. We speculate that repeated performance of the A15+15 or A30 protocols over several days or weeks may be necessary to observe such changes in hospitalized women with GDM. The significance observed in the pooled data was likely a statistical artifact driven by the aggregation of non-matched data points that individually lacked sufficient statistical power. Indeed, in the case of our study, a larger sample size may have provided statistical differences from time-point data.

Previous work in participants with T2DM demonstrated that split exercise, specifically 20 min of moderate-intensity CAE before and 20 min after lunch, resulted in lower glycemia in the hours following lunch than 40 min of CAE performed after the same meal (^17^). Van Dijk and cols. (^18^) found that 15-min bouts of slow-paced strolling (~100 bpm) after each main meal (totaling 45 min/day) over three days lowered postprandial glycemia in non-insulin-treated patients with T2DM, compared with a sedentary control condition in which participants spent the day seated on a couch, talking, reading, or watching television. In this line, our findings showed interesting partial glycemic benefits from interrupting the sedentary behavior commonly observed during hospitalization (extended time spent lying or sitting). These benefits were achieved using simple, very short (two 15-min bouts/day), low-intensity CAE (~105 bpm, ~56% HR_max_) in women with GDM. Moreover, because half of the participants used rapid-acting or intermediate-acting insulin, an additive effect between exogenous insulin and muscle contractility during the A15+15 protocol may have accelerated glucose uptake. Furthermore, pre-exercise CBG levels in the morning (~7.8 mmol/L) were higher than those before the afternoon exercise sessions (~6.3 mmol/L). This disparity was likely associated with exogenous insulin pharmacokinetics, specifically increased basal/bolus active insulin after lunch.

In the current study, eight of the nine participants with GDM exceeded the recommended upper fasting glucose limit of 5.1 mmol/L (^14^) in at least one experimental condition, although their glycated hemoglobin (HbA_1c_) levels (~5.4% or 36 mmol/mol) indicated normal baseline glycemic control (Table 1). In addition, it is not surprising that CAE exercise did not induce hypoglycemic episodes in the time points measured even in women under insulin therapy, as the cycling sessions were relatively short, and meals and snacks were served at regular intervals throughout the participants’ hospital stay.

Some limitations of our study must be acknowledged. First, the participant cohort was susceptible to selection bias, since the women who agreed to participate may have represented those with less complicated pregnancies, greater health awareness, or stronger commitment to lifestyle changes during their current pregnancy. Other relevant shortfalls of this pilot study include the relatively small sample size and the convenience sample, which partially limits generalizability. Despite the possible bias, a finger pulse oximeter was used to monitor HR during exercise sessions due to its ease of cleaning during the COVID-19 pandemic. In this regard, a recent study found no significant differences between HR values recorded by a finger pulse oximeter and a 12-lead ECG during low-intensity exercise (^19^). The contemporary need to standardize sensor technologies in clinical and sports settings remains critical to ensure robust data validity (^20^). Therefore, future research should incorporate responsive devices to accurately evaluate recovery and physiological load (^21^) after exercise sessions in women with GDM, in addition to mitigating risks and enhancing performance (^22^).

Other critical limitations of this pilot study included a potential “period effect” (e.g., adaptation to the hospital environment, reduced stress over time, sleep normalization, or the stabilization of diet and medication). Because the REST condition was strictly performed first, the study design could not rule out an order effect. Another significant issue was the absence of a continuous glucose monitoring system. Although continuous glucose monitoring measures interstitial glucose levels every few minutes, detects subtle physiological excursions, and provides detailed and valuable monitoring of time-course glycemic changes (^3^,^4^), it entails substantial costs. In our study, glucose levels were measured using a hand-held glucose meter, since capillary tests require minimal blood volume, are highly cost-effective, and reflect the pragmatic methods utilized outside rigorous research settings (^23^,^24^) in the conventional management of diabetes.

Despite these limitations, the preliminary data provide novel insights for the research field and exercise prescription for women with GDM. This population is generally more likely to engage in low-intensity exercise than moderate intensity exercise. In addition to the relatively short-duration patterns, our study evidenced that low-intensity CAE sessions (especially A15+15) may partially modulate glycemia following routine everyday meals (and not oral glucose tolerance tests). Because these findings are limited to the acute and subacute effects of the exercise session(s), future research is required to investigate the impact of regular low-intensity, short-duration aerobic exercise training on the glycemic parameters in this population.

In conclusion, although the non-randomized allocation of the control condition remained a critical methodological flaw in this work, low-intensity and relatively short-duration (15 min) aerobic exercise sessions performed twice a day effectively attenuated mean daily (pre- and postprandial) CBG levels in hospitalized women with GDM compared to a complete sedentary pattern during a hospital stay, suggesting its potential in clinical practice. This attenuation was only observed in the pooled data, as specific postprandial time points showed no significant differences. No significant differences in glycemic parameters were observed between the A30 and A15+15 protocols. These findings hold relevance for endocrinology research and broader healthcare practice, as the A15+15 regimen aligns with established physical activity guidelines and offers novel clinical insights to inform exercise prescriptions.

## Data Availability

the datasets used and/or analyzed during the current study are available from the corresponding author upon reasonable request.
